# Ferroptosis: a new mechanism of traditional Chinese medicine for cancer treatment

**DOI:** 10.3389/fphar.2024.1290120

**Published:** 2024-01-16

**Authors:** Jiahao Zhu, Peipei Shen, Yu Xu, Xiaojun Zhang, Qingqing Chen, Ke Gu, Shengjun Ji, Bo Yang, Yutian Zhao

**Affiliations:** ^1^ Department of Radiotherapy and Oncology, The Affiliated Hospital of Jiangnan University, Wuxi, Jiangsu, China; ^2^ Wuxi Clinical Cancer Center, Wuxi, Jiangsu, China; ^3^ Department of Radiotherapy and Oncology, The Affiliated Suzhou Hospital of Nanjing Medical University, Gusu School, Nanjing Medical University, Suzhou, Jiangsu, China

**Keywords:** traditional Chinese medicine, ferroptosis, function mechanism, clinical application, cancer

## Abstract

Ferroptosis, distinct from apoptosis, is a novel cellular death pathway characterized by the build-up of lipid peroxidation and reactive oxygen species (ROS) derived from lipids within cells. Recent studies demonstrated the efficacy of ferroptosis inducers in targeting malignant cells, thereby establishing a promising avenue for combating cancer. Traditional Chinese medicine (TCM) has a long history of use and is widely used in cancer treatment. TCM takes a holistic approach, viewing the patient as a system and utilizing herbal formulas to address complex diseases such as cancer. Recent TCM studies have elucidated the molecular mechanisms underlying ferroptosis induction during cancer treatment. These studies have identified numerous plant metabolites and derivatives that target multiple pathways and molecular targets. TCM can induce ferroptosis in tumor cells through various regulatory mechanisms, such as amino acid, iron, and lipid metabolism pathways, which may provide novel therapeutic strategies for apoptosis-resistant cancer treatment. TCM also influence anticancer immunotherapy via ferroptosis. This review comprehensively elucidates the molecular mechanisms underlying ferroptosis, highlights the pivotal regulatory genes involved in orchestrating this process, evaluates the advancements made in TCM research pertaining to ferroptosis, and provides theoretical insights into the induction of ferroptosis in tumors using botanical drugs.

## 1 Introduction

Cancer, a significant public health concern with substantial implications for global mortality and quality of life, poses a significant challenge as it remains a bereft of definitive curative interventions (Siegel et al., 2023). According to data provided by the World Health Organization, approximately 19.29 million new cancer cases were diagnosed worldwide in 2020, resulting in 9.96 million deaths related to cancer (Sung et al., 2021). Despite the potential clinical advantages associated with multimodal approaches to cancer treatment, the annual mortality rate of cancer patients continues to increase. The tumor initiation and progression cascade is a complex biological process.

Recently, ferroptosis was shown to play a crucial role in cancer development and treatment. In 2012, Stockwell introduced ferroptosis as a unique form of cell death that differs from apoptosis, necrosis, and autophagy. Ferroptosis is dependent on iron and characterized by the accumulation of lipid peroxidation caused by reactive oxygen species (Dixon et al., 2012). Ferroptosis is characterized by a significant reduction in mitochondrial size, heightened density of double membranes, absence or decrease in mitochondrial cristae, undamaged cell membranes, regular cell nucleus size, and dispersed chromatin (Wu et al., 2019). In terms of cellular morphology and function, ferroptosis does not exhibit typical necrotic features or classical apoptotic characteristics, nor does it form the enclosed double-membrane structures associated with autophagy. These two primary mechanisms trigger ferroptosis. The first is the endogenous pathway, which induces ferroptosis by inhibiting the activation of intracellular antioxidant enzymes, notably glutathione peroxidase 4 (GPX4). The second mechanism is the exogenous pathway, which initiates ferroptosis by blocking cellular membrane transporters such as system xcˉ or ferroportin. This pathway also activates transferrin and lactoferrin (Liu et al., 2022).

GPX4, dihydroorotate dehydrogenase (DHODH), and ferroptosis suppressor protein 1 (FSP1) represent the three main defense mechanisms against ferroptosis, whereas RSL3 and erastin are the two experimental compounds that have been identified to induce ferroptosis (Gao et al., 2022). GPX4 is the fourth member of the selenoprotein GPX family (Chen et al., 2021). In 2014, metabolomics studies revealed that GPX4 overexpression and knockdown modulated the lethality of 12 ferroptosis inducers (Yang et al., 2014). GPX4 is a crucial regulator of ferroptosis. GPX4 reduces the harmful effects of lipid peroxides and maintains homeostasis of the lipid bilayer through its catalytic activity. RSL3 ((1S,3R)-RSL3) functions as a GPX4 inhibitor by directly binding to and deactivating GPX4, which results in the build-up of lipid peroxides and initiation of ferroptosis (Chen et al., 2021). FSP1 potentially functions as an NADH oxidase and possesses the ability to inhibit ferroptosis through cysteine uptake. Erastin hinders the activity of the glutamate-cysteine antiporter system by regulating the equimolar exchange of intracellular glutamate and extracellular cysteine, leading to an inability to synthesize glutathione (GSH). This leads to the build-up of lipid peroxides, causing harm to proteins and membranes, and triggering cellular ferroptosis (Zhang et al., 2020). It has been discovered that the induction of ferroptosis could inhibit tumor cell growth via multiple targets and pathways (Zhang et al., 2018c). These new therapeutic strategies contribute to the development of novel cancer chemotherapy drugs and help overcome drug resistance in tumors (Doll et al., 2017).

Owing to its natural origins and medicinal properties, traditional Chinese medicine (TCM) has a rich historical usage globally for improving human health. TCM is characterized by diverse components, targets, and pathways, which provide special clinical benefits for tumor treatment. Recent clinical studies have provided compelling evidence that TCM can effectively extend the survival and enhance the quality of life of patients undergoing treatment for different types of cancer, including liver, gastric, and lung cancers (Li et al., 2022b; Hou et al., 2022). However, most TCM prescriptions lack clear targeting specificity and specific signaling pathways. This article comprehensively reviews the prevalence and underlying mechanisms of ferroptosis in cancer cells. Moreover, we review the current status of TCM in improving cancer therapeutic effects through various molecular mechanisms, mainly concerning amino acid, lipid, and iron metabolism. Additionally, this review summarizes the influence of TCMs on the tumor microenvironment by regulating ferroptosis.

## 2 Core mechanisms of ferroptosis

**FIGURE 1:**
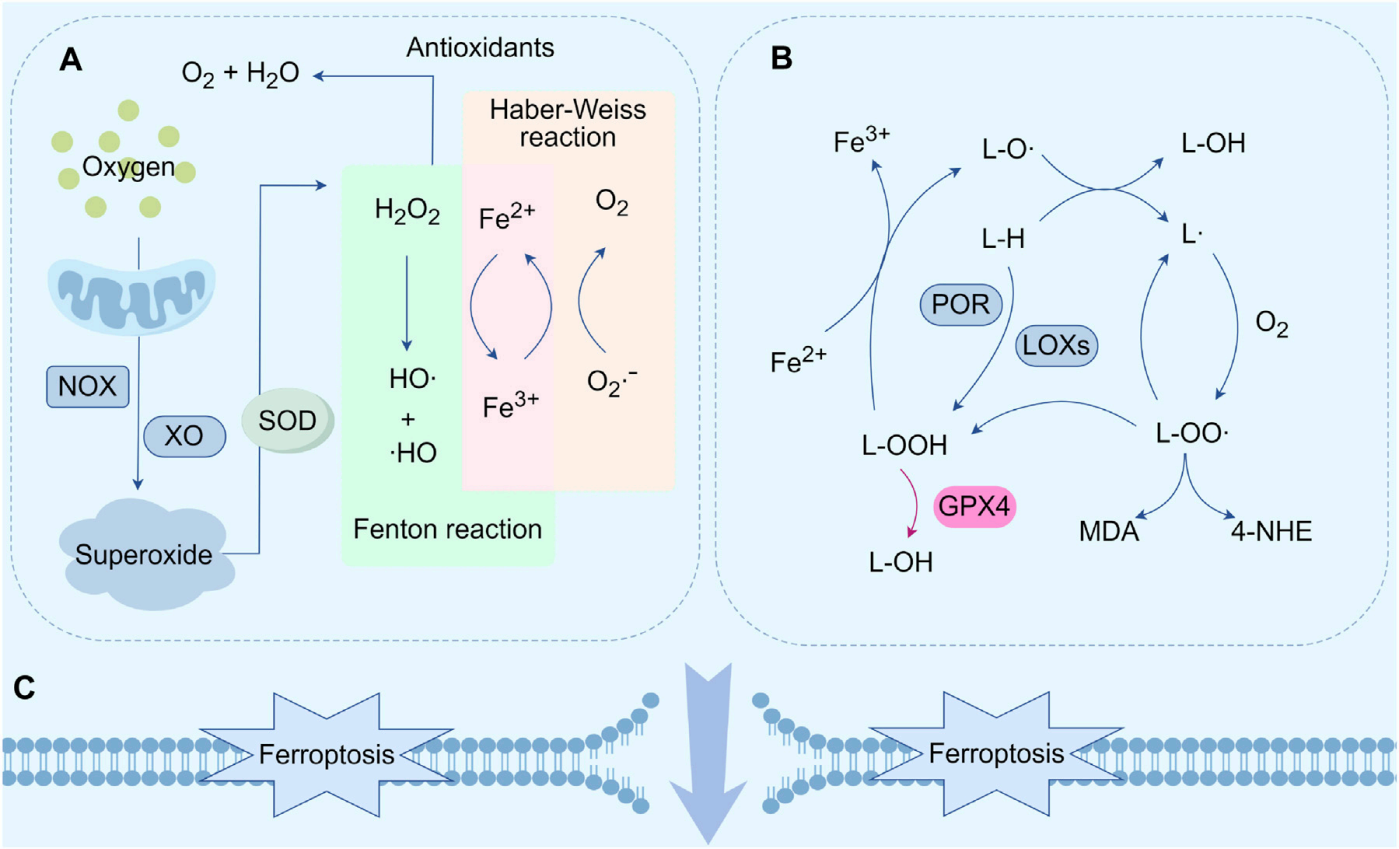
Core mechanisms of ferroptosis. **(A)** Generation of OH·. **(B)** Lipid peroxidation cycle and its termination. **(C)** Cell membrane rupture and ferroptosis. Abbreviations: NOX, nicotinamide adenine dinucleotide phosphate oxidase; XO, xanthine oxidase; O2·ˉ, superoxide radicals; SOD, superoxide dismutase; HO·, hydroxyl radicals; L-H, lipids; L·, lipid radicals; LO·, lipid free radicals; LOO·, lipid peroxy-free radicals; LOOH, lipids hydroperoxide; POR, cytochrome P450 reductase; LOXs, lipoxygenases; GPX4, glutathione peroxidase 4; 4-HNE, 4-Hydroxy-2-nonanal; MDA, malondialdehyde.

The oxidation of polyunsaturated fatty acids (PUFAs) on the lipid membrane by reactive oxygen species (ROS), such as superoxide anions (O_2_·ˉ), hydrogen peroxide (H_2_O_2_), hydroxyl radicals (OH·), and lipid hydroperoxide (L-OOH), leads to the formation of lipid peroxides. These peroxides damage cell membranes and trigger ferroptosis (Dixon et al., 2012). Free radical-driven lipid peroxidation is a hallmark of ferroptosis (Ge et al., 2021) (Figure 1).

The production of hydroxyl radicals (HO·) and peroxy radicals (RO·) is essential for initiating the lipid peroxidation process. Competitive deprivation of electrons from nicotinamide adenine dinucleotide phosphate (NADPH) and various pathways like mitochondrial electron transport chain (ETC), NADPH oxidase (NOX), and xanthine oxidase (XO) convert oxygen into O2·ˉ (Wen et al., 2013; Sakellariou et al., 2014; Yan et al., 2021). Superoxide is cleared by the enzyme superoxide dismutase 1 (SOD1), which catalyzes its dismutation into hydrogen peroxide (H_2_O_2_) and water (H_2_O), leading to decreased reactivity (Sullivan et al., 2014). The combination of glutathione peroxidase (GPX), peroxiredoxins (PRX), and catalase synergistically reduces H_2_O_2_ to H_2_O and oxygen (O_2_) (Mailloux, 2015). Fe^2+^ catalyzes the formation of hydroxyl radicals (OH·) from H_2_O_2_), which act as the foundation for the Fenton reaction, a process of free radical lipid peroxidation (Cheng et al., 2007; Pan et al., 2020). The Fenton reaction produces hydroxyl radicals, which then participate in the Haber-Weiss reaction to form a closed-loop cycle (Figure 1A).

Lipid peroxides are generated by the peroxidation of PUFAs by lipid alkoxy radicals (L-O ·), lipid peroxyl radicals (L-OO ·), and L-OOH (Conrad et al., 2018). Lipid peroxidation, catalyzed by lipoxygenases (LOXs), serves as the basis of ferroptosis and mainly includes three stages (Figure 1B). First, generation of carbon-centered lipid radicals (L·) through reactions between L-H and L-O·; second, production of L-OO· with L· and O_2_ through a positive feedback loop. Then new L· and L-OOH are produced by the reaction between L-OO· and another L-H. Additionally, L-OOH transforms into L-O· under the catalysis of iron. Finally, L-OO· converts into L-OH and O2, if the reaction with antioxidants is stopped (Feng et al., 2018; Zhao et al., 2022; Shi et al., 2023b). Notably, GPX4 is the only enzyme capable of decreasing hydrogen peroxide levels inside the cells and transforming L-OOH into L-OH, thus preventing lipid autoxidation reactions (Brigelius-Flohé and Maiorino, 2013).

The build-up of phospholipid hydroperoxides on cellular membranes is regarded as a defining characteristic that regulates the speed of ferroptosis. However, downstream pathways involved in ferroptosis remain unclear. Recent studies suggested three hypotheses regarding the regulation of ferroptosis by cellular activities (Figure 1C). According to molecular dynamics simulations, membrane oxidation, subsequent thinning, and an increase in curvature are facilitated by enhanced membrane curvature and a reduction in membrane thickness, enabling the penetration of oxidants into the membrane. Membrane damage is caused by an unregulated cycle of positive feedback, which is facilitated by the creation of pores and micellization (Agmon et al., 2018; Tang et al., 2019). Another theory proposes that ferroptosis is characterized by the creation of protein channels and the oxidation of polyunsaturated fatty acid fragments. The former disturbs the balance of ions, whereas the latter disturbs various cellular functions by harming the cell membranes and producing harmful substances. Cell swelling and death occur because of the ability of protein pores to facilitate unrestricted interactions with the surrounding environment (Magtanong et al., 2016). The final theory considers that 4-hydroxy-2-nonenal (4-HNE) be the ultimate product of lipid peroxidation derived from the conversion of malondialdehyde (MDA). The formation of covalent adducts with biomolecules diminishes the integrity of membranes and forms crosslinks, ultimately resulting in protein deactivation. This promotes cell membrane rupture and ferroptosis (Zhong et al., 2015).

## 3 Ferroptosis regulation in cancer

### 3.1 Regulation of TCM on ferroptosis through amino acid metabolism pathway

**FIGURE 2:**
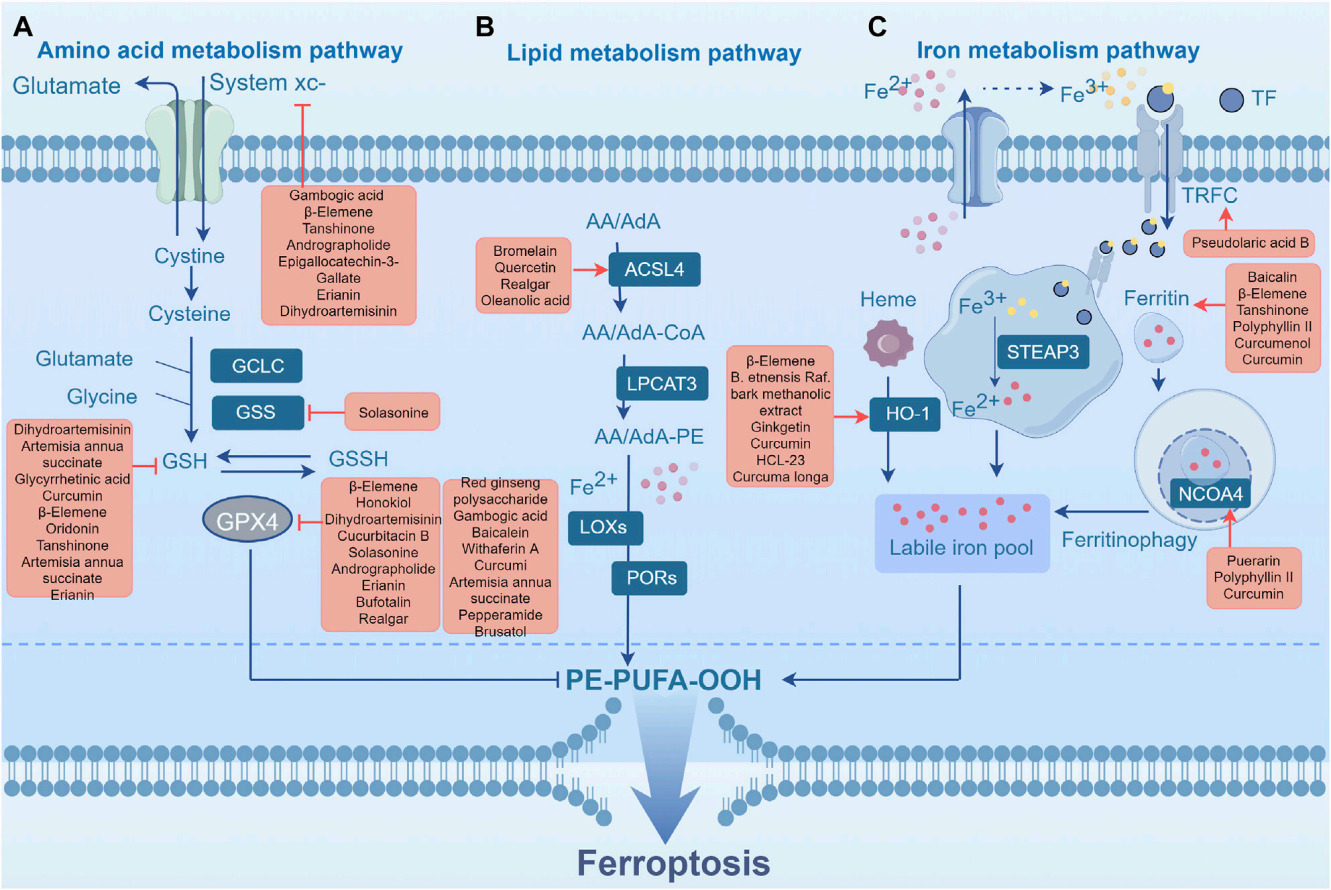
Ferroptosis metabolic pathway. **(A)** Amino acid metabolism pathway. **(B)** Lipid metabolism pathway. **(C)** Iron metabolism pathway. Abbreviations: GPX4, glutathione peroxidase 4; GCLC, glutamate-cysteine ligase catalytic subunit; GSH, glutathione; GSS, Glutathione synthetase; GSSH, glutathione counterpart; System xcˉ, Cystine-glutamate antiporter; POR, cytochrome P450 reductase; LOXs, lipoxygenases; LPCAT3, lysophosphatidylcholine acyltransferase 3; PL-PUFA-OOH, phospholipid hydroperoxides; ACSL4, acyl-CoA synthetase long-chain family member 4; AA, arachidonic acid; AdA, epinephrine; PE, phosphatidylethanolamine; CoA, coenzyme A; TF, transferrin; TFRC, transferrin receptor; HO-1, heme oxygenase-1; NCOA4, nuclear receptor coactivator 4; PL-PUFA-OOH, phospholipid hydroperoxides; STEAP3, Six-transmembrane epithelial antigen of the prostate 3.

The metabolism of cysteine and glutamate is closely related to the clearance of reactive oxygen species (ROS) and the activity of lipid peroxidation through the system xcˉ-GPX4 axis, which acts as a fundamental element in the defense mechanism against ferroptosis. The transsulfuration pathway and system xcˉ are the primary sources of Cys. The amino acid transporter protein system xcˉ consists of two subunits, namely, SLC7A11 (a member of the solute carrier family 7) and SLC3A2 (a member of the solute carrier family 3 member 2) (Sato et al., 1999). This transmembrane transporter, located on the phospholipid bilayer, facilitates the import of cysteine from the extracellular environment while simultaneously exporting glutamate in a 1:1 ratio (Conrad et al., 2012). Cysteine is then reduced to cysteine by thioredoxins or thioredoxin-related proteins, thereby fueling GSH synthesis. Glutathione (GSH) is formed by the combination of cysteine, glutamate, and glycine, catalyzed by γ-glutamylcysteine synthetase and glutathione synthetase. Cysteine is a crucial substrate for the synthesis of GSH and controls its rate (Liu et al., 2019). GSH is a necessary cofactor for GPX4 (glutathione peroxidase 4), a selenoprotein that contains a catalytic site for selenocysteine to exert its antioxidant function in eliminating lipid peroxides (Ingold et al., 2018). GSH deficiency impairs GPX4 function and lipid reactive oxygen species (ROS) accumulation, resulting in ferroptosis. GSH synthesis requires the presence of glutamate, another crucial substrate. The catalytic subunit of glutamate-cysteine ligase (GCLC) enables the initial stage of GSH production by catalyzing the connection between glutamate and cysteine. However, in the absence of sufficient cysteine, GCLC facilitates the production of γ-glutamyl peptides (γ-Glu-AAs), thereby removing surplus glutamate and preventing ferroptosis. In general, the metabolism of cysteine and glutamate influences cysteine uptake and GSH synthesis and then affects the activity of GPX4 (Figure 2A). Several TCMs influence this process to regulate ferroptosis in tumors and exert therapeutic effects.

Previous studies have demonstrated that various plant metabolites such as ursolic acid, oleanolic acid, Jiyuan oridonin A (JDA), Cucurbitacin B (CuB), Brucine, Pseudolaric acid B (PAB), Ginkgetin, β-elemene, and et al. can induce ferroptosis by intervening in the GSH/GPX4 pathway and then inhibit tumor growth (Wang et al., 2018; Chen et al., 2020a; Gao et al., 2020; Huang et al., 2021; Liu et al., 2021; Lou et al., 2021; Lu et al., 2021). Gao et al. discovered that *Actinidia chinensis Planch* (Actinidiaceae) (ACP) contains ursolic acid and oleanolic acid, the two primary anti-tumor metabolites. These metabolites enhance ROS accumulation by suppressing the expression of SLC7A11 and GPX4 in gastric cancer cells, ultimately inducing ferroptosis (Gao et al., 2020). JDA, an extract of Jiyuan Rabdosia rubescens, has also been observed to induce ferroptosis via similar mechanisms by downregulating GPX4 expression in gastric cancer cells (Liu et al., 2021). CuB, a triterpenoid molecule, strongly promotes the build-up of iron ions, depletes glutathione, and downregulates the expression of GPX4, leading to the excessive production of lipid peroxides (Huang et al., 2021). CuB initiates a multifaceted mechanism of ferroptosis in nasopharyngeal carcinoma cells, indicating its tumor-suppressive effect. Brucine, an indole alkaloid extracted from the seeds of *Strychnos nux-vomica* L. (Loganiaceae), activates endoplasmic reticulum stress in glioma cells, resulting in the upregulation of activating transcription factor 3 (ATF3) and its nuclear translocation (Lu et al., 2021). Subsequently, ATF3 promotes the accumulation of intracellular H_2_O_2_ by upregulating NOX4 and downregulating SLC7A11 and catalase. Ultimately, the ferroptosis induced by H_2_O_2_ regulated through the transferrin receptor, leads to cell death in glioma cells. PAB, a diterpenic acid obtained from the root and trunk bark of *Pseudolarix amabilis* (J. Nelson) Rehder (Pinaceae), induces ferroptosis in glioma cells via a similar pathway (Wang et al., 2018). Ginkgetin, a bioflavonoid from *Ginkgo biloba* L. (Ginkgoaceae) leaves, has been reported to increase cytotoxicity in small-cell lung cancer (NSCLS) treatment when combined with cisplatin (Lou et al., 2021). Administration of ginkgetin and cisplatin contributed to the suppression of SLC7A11 and GPX4 as well as the reduction of Nrf2/HO-1 in these NSCLS cells, disrupting redox homeostasis and resulting in the amplification of ginkgetin-induced ferroptosis and DDP-induced apoptosis. Chen et al. found that β-elemene, an active substance derived from *Curcuma longa* L. (Zingiberaceae), functions as a unique stimulates ferroptosis. The combination therapy of β-elemene and cetuximab suppresses GPX4 expression, inducing ferroptosis and inhibiting epithelial-mesenchymal transition (EMT) in colorectal cancer (CRC) cells with the KRAS mutation (Chen et al., 2020a). Dihydroisotanshinone I, obtained from Danshen, *Salvia miltiorrhiza Bunge* (Lamiaceae), triggers ferroptosis by inhibiting GPX4 expression in breast cancer (Lin et al., 2019). In addition, the derivatives of various plant metabolites can target the GSH/GPX4 pathway and induce ferroptosis. Parthenolide, a prominent germacrane type sesquiterpene lactone, has shown anticancer activity (Ghantous et al., 2013). Its derivative DMOCPTL induces ferroptosis and early growth response-mediated apoptosis in breast cancer cells through the ubiquitination of GPX4 (Ding et al., 2021). ALZ003, an analog of curcumin, triggers ferroptosis in glioblastoma cells by simultaneously inducing GPX4 downregulation and lipid peroxidation (Chen et al., 2020b).

Various metabolites and derivatives have been investigated for their ability to induce ferroptosis in various types of cancers. Ursolic acid and oleanolic acid in ACP, as well as JDA from Jiyuan Rabdosia rubescens, promote ferroptosis in gastric cancer cells by suppressing SLC7A11 and GPX4 expression. CuB, Brucine, PAB, ginkgetin, β-elemene, Danshen DMOCPTL, and ALZ003 were also identified as inducers of ferroptosis in different cancer types through various mechanisms targeting proteins involved in redox homeostasis. These findings highlight the potential of these metabolites and their derivatives in the development of novel therapeutic strategies for cancer treatment. Currently, most study concerning TCM on the regulation of ferroptosis in the amino acid metabolic pathway have focused on the observation of specific molecules after active ingredient intervention, especially GPX4 and SLC7A11. Compared with the lipid and iron metabolism pathways, the amino acid metabolism pathway has been the most studied; however, few studies have investigated the specific upstream and downstream regulation of TCM. Further mechanistic studies are warrant.

### 3.2 Regulation of TCM on ferroptosis through lipid metabolism pathway

Acyl-CoA synthetase long-chain family member 4 (ACSL4) and lysophosphatidylcholine acyltransferase 3 (LPCAT3) play crucial roles in promoting lipid peroxidation during ferroptosis. Long-chain PUFAs such as arachidonic acid (AA) and adrenic acid (AdA) exist primarily in their free forms. The derivatized AA-CoA or AdA-CoA is combined with CoA through ACSL4, and subsequently subjected to additional modifications by LPCAT3 to generate membrane phosphatidylethanolamine (AA-PE or AdA-PE) (Yu et al., 2023). PE-AA is a PUFA that can be catalyzed by LOX15 and Fe^2+^ to generate oxidized phospholipid ox-PE, thereby participating in the initiation of ferroptosis (Shimada et al., 2016). Downregulation of ACSL4 suppressed ferroptosis in several cancer cells. ACSL4 expression is also regulated by radiotherapy and immunotherapy (Doll et al., 2017; Mbaveng et al., 2018). Loss of LPCAT3 resulted in a notable decrease in AA levels within the cellular membranes, along with the accumulation of lipid droplets in the cytoplasm. This ultimately leads to the death of mice and damage to their intestinal cells, highlighting the importance of LPCAT3 in cell viability (Zhang et al., 2018b). According to Shah et al., enzymatic lipid peroxidation reactions, rather than non-enzymatic lipid peroxidation, play a crucial role in achieving the critical threshold of phospholipid hydroperoxides (PLOOH) for ferroptosis (Shah et al., 2018; Zheng and Conrad, 2020). Given that the neurological system harbors a significant abundance of PUFAs, ferroptosis induction may show promising efficacy in brain tumor treatment (Bano et al., 2022). Previous studies have demonstrated that the ferroptosis inducers, erastin and sorafenib, exert potent cytotoxic effects (Gagliardi et al., 2020). Currently, temozolomide remains the primary chemotherapeutic agent for glioblastoma; however, patients commonly exhibit poor treatment response and survival after treatment. Buccarelli et al. unveiled the capacity of quinacrine, a compound capable of traversing the blood-brain barrier, to enhance the vulnerability of glioblastoma stem-like cells to temozolomide through an iron-dependent form of programmed cell death known as ferroptosis (Buccarelli et al., 2018). This observation implies that induction of ferroptosis in GSCs holds significant promise as a novel and crucial therapeutic strategy for glioblastoma management. Several TCMs regulate ferroptosis by influencing lipid metabolism in tumors and exerting therapeutic effects.

Curcumin, a polyphenol extracted from turmeric plants, upregulates ACSL4 expression and inhibits SLC7A11 and GPX4, leading to iron overload, GSH depletion, and lipid peroxidation, ultimately triggering ferroptosis in NSCLC cells (Tang et al., 2021). Bromelain is a combination of proteolytic enzymes (proteases) isolated from *Ananas comosus* (L.) Merr. (Bromeliaceae) stems and was found to effectively induce ferroptosis in Kras-mutant colorectal cell lines by regulating the levels of ACSL-4 compared to wild-type cells (Park et al., 2018). Oleanolic acid (OA) is a pentacyclic triterpene that occurs naturally in the leaves, fruits, and seeds of plants and possesses diverse biological functions, such as antioxidant, anti-inflammatory, and anticancer effects (Pollier and Goossens, 2012; Kashyap et al., 2016; Zhang et al., 2018a). OA can increase oxidative stress levels and the concentration of divalent iron ions as well as the expression of iron death-related proteins and ACSL4. After knockdown of ACSL4 expression in cervical cancer cells, the anticancer effect of oleanolic acid was counteracted, resulting in a decrease in reactive oxygen species and GPX4 levels, suggesting that OA activates ferroptosis in cervical cancer cells by promoting ACSL4 expression (Jiang et al., 2021). Methanolic extract of *Betula etnensis* Raf. (Betulaceae) induces ROS generation and reduces antioxidant cell defense, leading to an imbalance in the cellular redox state and ferroptosis. This effect leads to a significant increase in lipid peroxidation and heme oxygenase-1 (HO-1) activity, further exacerbating colon cell death (Malfa et al., 2019). These metabolites or extracts trigger ferroptosis mainly by targeting ACSL4 in the lipid metabolism pathway, which may improve the therapeutic effects on cancer when combined with chemotherapy or targeted therapy.

Curcumin, artemisinin, bromelain, oleanolic acid, and the methanolic extract of *Betula pendula subsp. pendula* (Betulaceae). Showed ferroptosis-inducing effects in different cancer cell types. These compounds target key regulators of ferroptosis, mainly ACSL4, which promotes lipid peroxidation. By modulating these pathways, TCMs offer potential adjuncts to conventional cancer therapies, thereby enhancing their effectiveness. Further research on their mechanisms and therapeutic applications is required.

### 3.3 Regulation of TCM on ferroptosis through iron metabolism pathway

The regulatory network of ferroptosis relies heavily on iron ions, mainly via the Fenton reaction and the activation of enzymes that contain iron. This process not only supplies energy to cells, but also simultaneously produces a substantial quantity of lipid peroxide (Stockwell et al., 2017). Iron metabolism involves iron absorption, transport, storage, and utilization. Macrophages, which phagocytize and recycle iron from senescent red blood cells, are the main source of iron. Transferrin (TF) and its receptor (TFRC) mediate iron import from the extracellular transferrin into cells. These serve as key regulatory factors and specific biomarkers of ferroptosis (Gao et al., 2015). TF binds to TFRC and is transported to the lysosomes, where it releases Fe^3+^. Subsequently, the Six-Transmembrane Epithelial Antigen of the Prostate 3 (STEAP3) reduces Fe^3+^ to Fe^2+^, which is then released into the cytoplasmic labile iron pool (LIP) via divalent metal transporter 1 (DMT1) (Zhao et al., 2021). Additionally, facilitation of ferritinophagy by Nuclear Receptor Coactivator 4 (NCOA4) results in the production of a substantial quantity of Fe^2+^ in the LIP. Fe^2+^ participates in iron-mediated processes, or enters the mitochondria to serve as a cofactor for the synthesis of various enzymes (Khalil et al., 2017). Normally, cancer cells employ iron via the NFS1 (Cysteine Desulfurase)-ISCU (Iron-Sulfur Cluster Assembly Enzyme)-CISD1/2 (CDGSH Iron-Sulfur Domain Protein 1 and 2) pathway to hinder lipid peroxidation and ferroptosis in mitochondria. Nevertheless, an overwhelming surplus of Fe^2+^ in the mitochondria can result in the deactivation of enzymes, disruption of iron metabolism, and ferroptosis (Yuan et al., 2016; Kim et al., 2018; Fang et al., 2019). Ferroptosis is regulated by decreased transferrin levels. In human pancreatic cancer cells, NEDD4L the Nedd4-like E3 Ubiquitin Protein Ligase) mediates the ubiquitination and degradation of lactoferrin, a member of the transferrin family, leading to ferroptosis induction (Wang et al., 2020b). Abnormalities in iron metabolism-related genes contribute to ferroptosis in cancer cells. Nrf2 regulates iron metabolism through HO-1. Overactivation of HO-1 can trigger the breakdown of heme into Fe^2+^, resulting in iron ion toxicity. Nevertheless, a moderate increase in HO-1 expression can enhance its protective properties by boosting its antioxidant functions (Hassannia et al., 2018). In theory, each stage of iron metabolism provides potential opportunities for drug-induced ferroptosis and the development of anti-tumor strategies.

Dihydroartemisinin (DHA), a derivative of artemisinin, induces ferroptosis in acute myeloid leukemia cells via the AMPK/mTOR/p70S6K signaling pathway (Du et al., 2019). It induces ferritinophagy, accelerates ferritin degradation, increases LIP, promotes ROS build-up in cells, and ultimately leads to ferroptotic cell death. Iron-dependent cell death induced by DHA has also been observed in head and neck carcinoma cells (Lin et al., 2016). Wang et al. found that vitamin C significantly activates ferroptosis by inhibiting the growth of anaplastic thyroid cancer (ATC) cells. This effect is primarily characterized by GPX4 inactivation, reactive oxygen species (ROS) accumulation, and iron-dependent lipid peroxidation. Research suggests that ferritin degradation is induced after treatment with vitamin C, leading to the release of free iron. Excess Fe triggers ROS generation via the Fenton reaction. ROS and iron mediate a positive feedback loop, sustaining lipid peroxidation, and ultimately resulting in iron-dependent cell death in ATC cells (Wang et al., 2021). 6-Gingerol, a phenol extracted from *Zingiber officinale* Roscoe (Zingiberaceae), suppresses the growth of lung cancer cells by inhibiting USP14 and subsequently regulating autophagy-dependent ferroptosis (Tsai et al., 2020).

DHA and vitamin C have been identified as inducers of ferroptosis in cancer cells. DHA induces ferritin degradation, increases LIP levels, promotes ROS accumulation, and triggers ferroptosis Vitamin C inhibits GPX4 activity leading to ROS accumulation and iron-dependent lipid peroxidation. Both botanical drugs are potential therapeutic agents for specific cancer types. Additionally, 6-Gingerol inhibits lung cancer cell growth by targeting USP14 and regulating autophagy-dependent ferroptosis. These findings highlight the role of iron metabolism in ferroptosis and the potential of targeting this pathway for anti-tumor strategies.

### 3.4 Regulation of TCM on ferroptosis through other pathways

In addition to the main regulatory pathways of ferroptosis mentioned above, ferroptosis is also regulated by other pathways, including voltage-dependent anion channels in the mitochondria, the P53-SAT1-ALOX15 pathway, and P53/SLC7A11 (Zhao et al., 2022; Shi et al., 2023b). A study by Xie et al. demonstrated that robustaflavone, isolated from Selaginella trichoclada, triggered iron-dependent cell death in breast cancer cells by enhancing the expression of voltage-dependent anion channel 2 (VDAC2) and downregulating Nedd4 E3 ubiquitin ligase, resulting in the generation of lipid peroxidation and ROS (Xie et al., 2021). OU et al., found that SAT1, a direct target gene of p53, is involved in the regulation of iron-dependent cell death. P53 can activate SAT1, which, in turn, regulates arachidonate 15-lipoxygenase (ALOX-15), inducing cellular lipid peroxidation and ROS production, ultimately leading to ferroptosis (Ou et al., 2016). Huang et al. reported that lung cancer cells treated with erastin significantly increase the expression of p53 while decreasing the expression of SLC7A11, subsequently inhibiting system xcˉ activity, leading to a reduction in cystine uptake and decreased synthesis of GSH, further suppressing the activity of GPX4, ultimately inducing ferroptosis in these cells (Huang et al., 2018). Therefore, the role of p53 in the regulation of ferroptosis needs to be considered.

## 4 TCM and ferroptosis in tumor microenvironment

**FIGURE 3:**
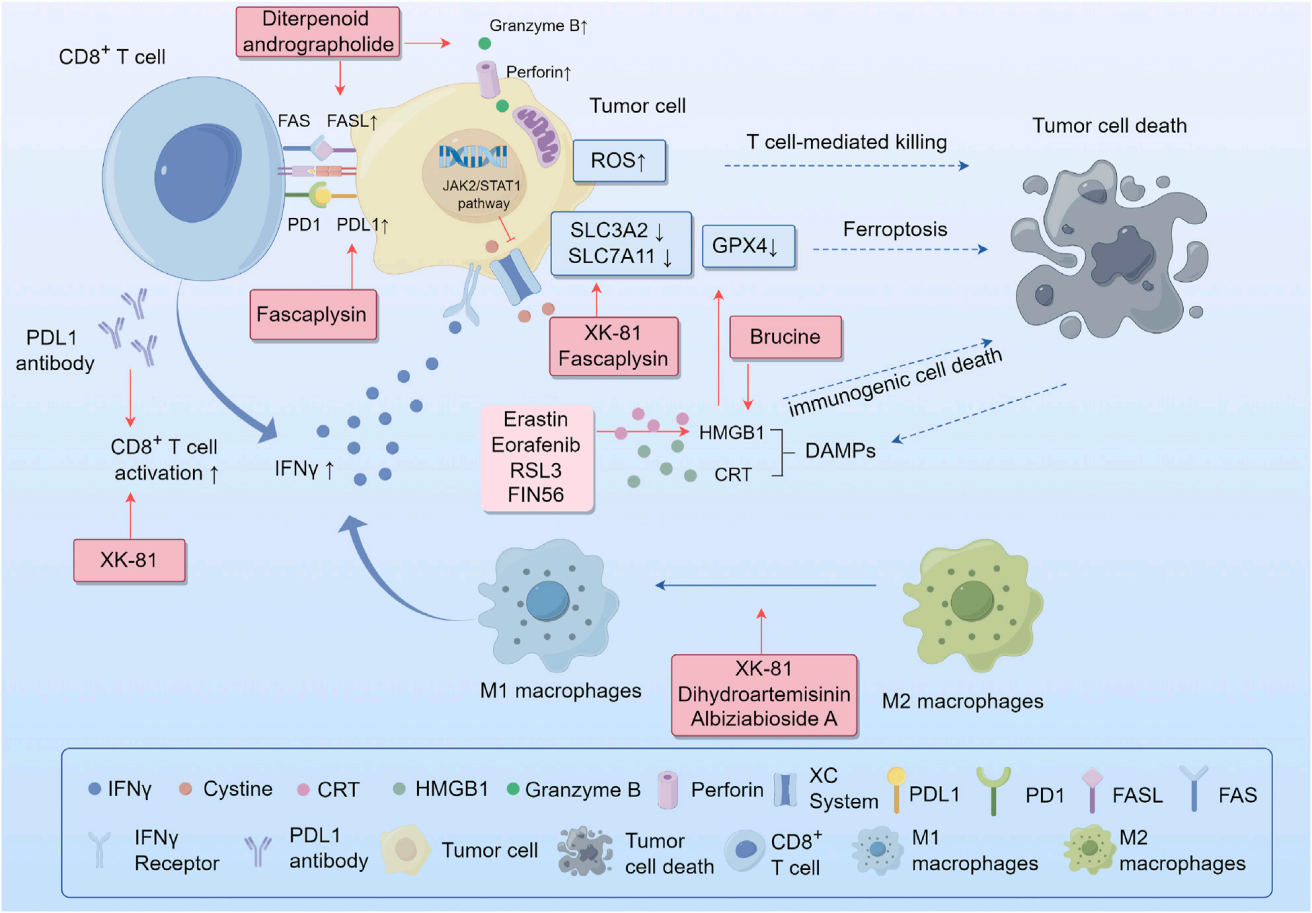
Synergistic interactions between ferroptosis and antitumor immunity. Abbreviations: IFNγ, interferon gamma; System xcˉ, cystine-glutamate antiporter; SLC3A2, solute carrier family 3 member 2; SLC7A11, cystine/glutamate antiporter solute carrier family 7 member 11; ROS, reactive oxygen species; GSH, glutathione; PD-1, programmed death-1; PD-L1, programmed death-ligand 1; DAMPs, damage-related molecular patterns; CRT, calreticulin; HMGB1, high-mobility group protein B1.

Recent breakthroughs in immunotherapy have led to a new era in cancer treatment. Immunotherapies for tumors, such as antibodies, cytokine immune checkpoint inhibitors, and cancer vaccines have demonstrated clinical efficacy and are promising strategies against cancer (Sharma et al., 2017). However, not all patients benefit equally from immunotherapy because of the multiple immune evasion mechanisms that develop in tumors, including inhibitory immune checkpoint expression, poor immune cell infiltration, and the release of immunosuppressive molecules (Zhang et al., 2020). Tumors can escape immune surveillance and attack the host immune system to establish, develop, migrate, and relapse. Developing new strategies to overcome these tolerance mechanisms is crucial to improve the effectiveness of immunotherapy. Several studies have revealed the synergistic interactions between ferroptosis and anti-tumor immunity regulated by TCM (Figure 3).

### 4.1 Alteration of immune checkpoints

Programmed death-1 (PD-1) and programmed death-ligand-1 (PD-L1) inhibitors are the most widely used anti-tumor immunotherapeutic drugs in clinical practice. The binding of PD-L1 to PD-1 on the surface of T cells causes the exhaustion of T cells. However, the use of PD-1/PD-L1 inhibitors to block this interaction restores the ability of CD8^+^ T cells to effectively eliminate cancerous cells. Recent studies have demonstrated that plant metabolites regulate immune checkpoint receptors to enhance immune response pathways. Fascaplysin, a bis-indole alkaloid isolated from the marine sponge *Fascaplysinopsis* Bergquist sp., has shown effective anticancer activity. Lou et al. demonstrated that fascaplysin induces ferroptosis and enhanced the expression of PD-L1, leading to increased responsiveness to anti-PD-L1 immunotherapy in NSCLS (Luo et al., 2022). Moreover, it has been reported that immunotherapy-activated CD8^+^ T cells can boost lipid peroxidation specific to ferroptosis in cancer cells, which ultimately leads to the improved effectiveness of immunotherapy against tumors. The release of interferon gamma (IFNγ) from CD8+T cells reduces the expression of SLC3A2 and SLC7A11, through JAK2/STAT1 pathway. This reduction hinders the absorption of cysteine by cancer cells, leading to lipid peroxidation and ferroptosis in these cells (Wang et al., 2019). These studies suggest that the positive synergistic interactions between Chinese medicine and checkpoint inhibitors further enhance the therapeutic efficacy of cancer immunotherapy. This synergistic effect has also been observed for andrographolides. Liu et al. reported that the diterpenoid andrographolide derived from *Andrographis paniculata* (Burm.) Nees (Acanthaceae), when used with an anti-PD1 antibody, enhances the activity of CD4^+^ and CD8^+^ T cells. This was demonstrated by substantial tissue infiltration, increased secretion of IFN-γ, and improved expression of molecules associated with cytotoxic T cells, such as FasL, perforin, and Granzyme B. Consequently, there was a significant reduction in the tumor load (Liu et al., 2020b). Moreover, some TCMs directly downregulate immune checkpoints. Baicalein and its conjugate baicalin are metabolites of *Scutellaria baicalensis* Georgi (Lamiaceae). Ke et al. found that these two flavonoids reduced STAT3 activity, leading to a decrease in IFN-γ-induced PD-L1 expression. As a result, T cell activity is restored, enabling the killing of hepatocellular carcinoma cells (Ke et al., 2019). Plant metabolites such as ferroptosis activators, combined with immune checkpoint inhibitors, may provide a promising strategy for cancer treatment.

### 4.2 Regulation of macrophage polarization

Tumor-associated macrophages (TAM) are one of the major types of immune cells that infiltrate tumors and are typically classified into two distinct subtypes with opposing functions: classically activated M1 macrophages and alternatively activated M2 macrophages. The former is generally involved in anti-tumor functions including direct cytotoxicity and antibody-dependent cell-mediated cytotoxicity (ADCC) to kill tumor cells. Conversely, the latter promotes tumor initiation and metastasis, secretes pro-angiogenic factors and immunosuppressive cytokines, and effectively inhibits the effector functions of T cells and M1 macrophages. Both M1 and M2 macrophages exhibit high plasticity, enabling them to switch between one another in response to alterations in the tumor microenvironment or therapeutic measures (Pan et al., 2020). Ferroptosis can trigger an inflammatory response to specific necrosis to regulate macrophage polarization in the tumor microenvironment (Bebber et al., 2020). Enhancing the M1/M2 macrophage ratio by developing an anticancer macrophage innate transformation therapy is a promising strategy for tumor treatment. Li et al. found that DHA-triggered ferroptosis in TAMs leads to DNA damage, which can activate downstream NF-κB signaling and remodel TAMs into an M1 phenotype in lung cancer (Li et al., 2022a). Another study showed that XK-81, a metabolite of *Leathesia nana* (Chordariaceae), decreased SLC7A11 and GPX4 expression and induced ferroptosis in tumor tissues. Moreover, XK-81 improves the function of CD8^+^ T cells and NK cells while simultaneously increasing the ratio of M1/M2 macrophages (Sun et al., 2023). Albiziabioside A (AlbA), an oleanane triterpenoid saponin, was extracted from the aerial parts of *Albizia inundata* (Mart.) Barneby and Grimes (Fabaceae) can also regulate macrophage polarization by eliminating M2-TAMs via triggering iron-dependent cell death (Wei et al., 2019).

### 4.3 Release of damage-related molecular patterns (DAMPs)

The release of DAMPs by various forms of cell death can result in the maturation of dendritic cells, reciprocal activation of CD8^+^ T cells, generation of IFN-γ, and the establishment of a positive loop between the immune and inflammatory responses (Yu et al., 2023). Phagocytosis of tumor cells undergoing ferroptosis is triggered by three important DAMPs in bone marrow-derived dendritic cells: calreticulin (CRT), high-mobility group protein B1 (HMGB1), and adenosine triphosphate (ATP) (Turubanova et al., 2019). HMGB1 has been reported to play a crucial role in maintaining the structure and function of chromosomes and is released by ferroptotic tumor cells in an autophagy-dependent manner. (Tao et al., 2017; Chen et al., 2022). Several ferroptosis agonists, including erastin, sorafenib, RSL3, and FIN56, could promote this release to activate macrophages, secrete CXCL1, TNF, and IFN-β, and contribute to immunogenic cell death (ICD) (Wen et al., 2019). Brucine, a ferroptosis activator, can trigger CRT exposure and release HMGB1 to induce ICD in cancer cells (Ishimwe et al., 2020). Wiernicki et al. found that a suppressive immune response occurs after the downregulation of GPX4 expression in tumor cells (Wiernicki et al., 2022). This is due to the upregulation of PD-L1 in tumor cells and the infiltration of myeloid-derived suppressor cells (MDSC) mediated by HMGB1, which counteracts the infiltration of cytotoxic CD8+T cells dependent on CXCL10 (Wen et al., 2019). However, the combination of withaferin A, a ferroptosis-inducing plant metabolite, and immunotherapy significantly extended the overall survival of a tumor mouse model (Conche et al., 2023).

## 5 Limitations and perspectives

**FIGURE 4:**
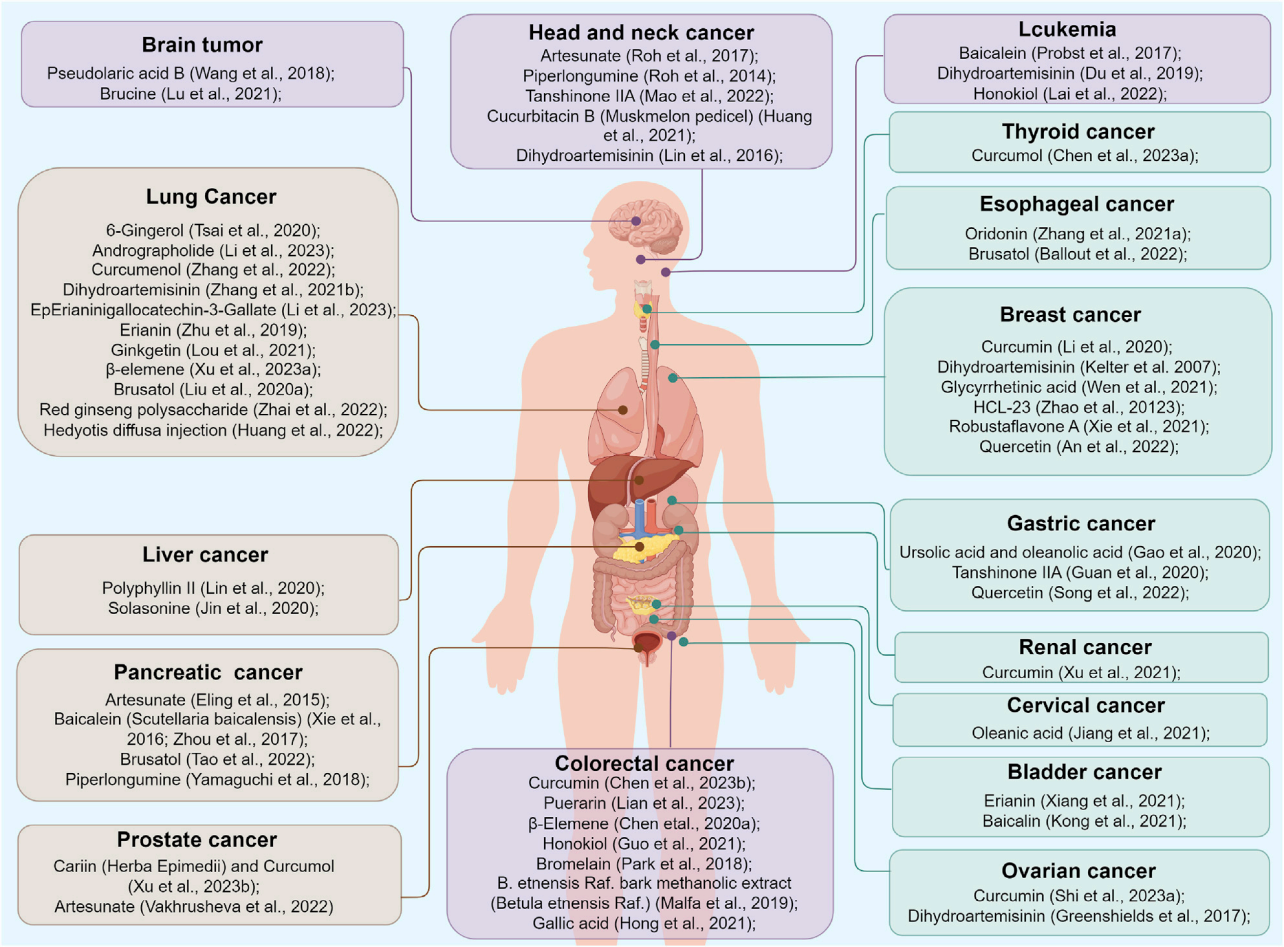
Various plant metabolites and derivatives inhibiting tumor cells growth by triggering ferroptosis.

Although recent research has greatly enhanced our understanding of ferroptosis, its underlying mechanisms have not been fully elucidated. First, ferroptosis is mainly identified by observing mitochondrial morphology, ROS levels, and SLC7A11 and GPX4 expression; no specific biomarkers for ferroptosis have been defined. Therefore, the actual frequency of ferroptosis in cancer remains unknown. Secondly, ferroptosis contributes to killing tumor cells while harming normal cells, thus exhibiting a dual nature. Additionally, the regulation of botanical drugs that target multiple ferroptosis pathways in clinical practice is complicated. Piperlongumine induces ferroptosis in both cancer and normal cells by promoting antioxidation (Tripathi et al., 2020). Exploring specific targets that trigger ferroptosis solely in tumor cells is an important direction for further research. Nanodelivery systems provide an intelligent platform to codeliver different therapeutic drugs in precise proportions to tumor tissues and selectively release them within cancer cells (Pacardo et al., 2015). Third, the relationship between ferroptosis and autophagy needs to be re-examined. Recently, ferroptosis has been recognized as an autophagic cell death process. Autophagy plays a crucial role in inducing ferroptosis via the selective autophagy of ferritin, known as ferritinophagy, and ROS generation. Interactions between ferroptosis and other cell death regulatory mechanisms, including apoptosis and necroptosis, have also been observed to varying degrees. The network of various mechanisms regulating ferroptosis and these cell death modes remains uncertain and warrants further study to be well elucidated. Fourth, significant progress has been made in the study of the preceding and intermediate routes of ferroptosis. Although studies on downstream signaling molecules in ferroptosis are limited, current research suggests that membrane collapse triggered by lipid peroxidation is the primary factor leading to ferroptosis (Bebber et al., 2020). Further investigations are needed to determine whether other effector molecules come into play after excessive lipid peroxidation, thereby accelerating iron-dependent cell death. Additionally, there is an ongoing debate about whether mitochondrial malfunction alone can initiate ferroptosis, and whether the impact of mitochondrial activity on ferroptosis varies depending on the environment. Fifth, in terms of the immune tumor environment, ferroptosis is a double-edged sword. As mentioned above, ferroptosis can elicit a host anticancer immune response, but can also lead to immunosuppression and antigen tolerance. The relationship between ferroptosis and immunotherapy remains in the primary stages of exploration and requires further research to elucidate the molecular mechanisms that provide opportunities for designing new therapeutic interventions. Sixth, ferroptosis-implicit tumor suppression may be weakened in metastatic tumor cells. Tumor cells enhance their capacity to metabolize fatty acids and develop a protective layer of oleic acid molecules on their surface through the action of ACSL3, enabling them to adapt to a lipid-rich environment in the lymph nodes (Ubellacker et al., 2020). However, the inhibitory effect of ferroptosis inducers on metastatic tumor cells is unknown. Finally, investigations into botanical drugs that modulate ferroptosis in cancer remain limited. Further research is warranted to provide high-quality evidence on the mechanisms by which plant metabolites and derivatives counteract tumors through ferroptotic interactions, which may provide new strategies for anticancer treatment. Figure 4 summarizes the various plant metabolites and derivatives that inhibit tumor cell growth by triggering ferroptosis. Table 1 shows the molecules and mechanisms of traditional Chinese medicine in the treatment of ferroptosis.

**TABLE 1:** Molecules and mechanisms of traditional Chinese medicine in the intervention of ferroptosis.

Metabolites and derivatives	Sources	Cancer	Mechanisms	Effects	References
Erianin	*Dendrobium chrysotoxum* Lindl. (Orchidaceae)	Bladder cancer	NRF2 inactivation	Promoting the accumulation of lethal lipid-based ROS and the depletion of GSH	Xiang et al. (2021)
Baicalin	*Scutellaria baicalensis* Georgi (Lamiaceae)	Bladder cancer	Inducing FTH1 dependent manner	ROS accumulation and intracellular chelate iron enrichment	Kong et al. (2021)
Curcumin	*Curcuma longa* L. (Zingiberaceae)	Breast cancer	Upregulating HO-1 expression	Accumulation of intracellular iron, reactive oxygen species, lipid peroxides, and malondialdehyde, while glutathione levels were significantly downregulated	Li et al. (2020)
Dihydroartemisinin	Artemisinin; *Artemisia annua* L. (Asteraceae)	Breast cancer	Inducing ABCB6 expression	Dysregulation of iron homeostasis	Kelter et al. (2007)
Glycyrrhetinic acid	*Glycyrrhiza uralensis* Fisch. ex DC. (Fabaceae)	Breast cancer	Activating NADPH oxidases and iNOS, and decreasing GSH and GPX activity	Aggravating lipid peroxidation	Wen et al. (2021)
HCL-23	Curcumol; *Curcuma zedoaria* (Christm.) Roscoe (Zingiberaceae)	Breast cancer	Upregulation of HO-1 expression	Increasing ROS, labile iron pool, and lipid peroxidation levels	Zhao et al. (2023)
Quercetin	Plants and vegetables	Breast cancer	Upregulation of TFEB and LAMP-1 expression	Promoted the degradation of ferritin and the release of ferric ions	An et al. (2022)
Robustaflavone A	*Selaginella siamensis* Hieron.(Selaginellaceae)	Breast cancer	Enhancing the expression of VDAC2 channels and reducing the expression of Nedd4 E3 ubiquitin ligase	Lipid peroxidation and ROS production	Xie et al. (2021)
Oleanolic acid	Plants and vegetables	Cervical cancer	Promoting ACSL4 expression	Increasing in ROS levels and GPX4	Jiang et al. (2021)
Curcumin	*Curcuma longa* L. (Zingiberaceae)	Colorectal cancer	Suppressing the PI3K/Akt/mTOR pathway	Downregulating GSH, SLC7A11, and GPX4, while Increasing levels of iron, malondialdehyde, and ROS.	Chen et al. (2023b)
Gallic acid	Plants and vegetables	Colorectal cancer	Inhibiting SIGMAR1 and activating ATF4	Reactive oxygen species (ROS) accumulation and intracellular chelate iron enrichment	Hong et al. (2021)
Puerarin	*Pueraria montana* var. *lobata* (Willd.) Maesen and S.M.Almeida ex Sanjappa and Predeep (Fabaceae)	Colorectal cancer	Triggering NCOA4 upregulation	Ferritinophagy	Lian et al. (2023)
β-Elemene	*Curcuma longa* L. (Zingiberaceae)	Colorectal cancer	Upregulation of HO-1 and transferrin, and downregulation of negative regulatory proteins for ferroptosis (GPX4, SLC7A11, FTH1, glutaminase, and SLC40A1)	Inducing iron-dependent ROS accumulation, GSH depletion, lipid peroxidation	Chen et al. (2020a)
Honokiol	*Magnolia officinalis* Rehder and E.H.Wilson (Magnoliaceae)	Colorectal cancer	Decreasing the activity of GPX4	Increasing ROS and Fe2+ levels	Guo et al. (2021)
Bromelain	*Ananas comosus* (L.) Merr. (Bromeliaceae)	Colorectal cancer	Through upregulated expression of ACSL-4	Lipid peroxidation	Park et al. (2018)
B. etnensis Raf. bark methanolic extract	*Betula pendula subsp. pendula* (Betulaceae)	Colorectal cancer	Inducing HO-1 expression	ROS production	Malfa et al. (2019)
Oridonin	*Isodon rubescens* (Hemsl.) H.Hara (Lamiaceae)	Esophageal cancer	Inhibiting the gamma-glutamyl cycle	Reducing GSH/GSSG, and decreasing the enzymatic activity of GPX4	Zhang et al. (2021a)
Brusatol	*Brucea javanica* (L.) Merr. (Simaroubaceae)	Esophageal cancer	Inhibiting NRF2 transcriptional activity and downregulating the NRF2 expression	Lipid peroxidation	Ballout et al. (2022)
Ursolic acid and oleanolic acid	*Actinidia chinensis Planch* (Actinidiaceae)	Gastric cancer	Downregulating the expression levels of Vimentin protein and Snail protein	Increasing the accumulation of ROS via inhibited GPX4 and SLC7A11 proteins	Gao et al. (2020)
Tanshinone IIA	*Salvia miltiorrhiza Bunge* (Lamiaceae)	Gastric cancer	Through p53-mediated SLC7A11 downregulation	Decreasing intracellular GSH level and cysteine level and increasing intracellular ROS level	Guan et al. (2020)
Quercetin	Plants and vegetables	Gastric Cancer	Affecting the JAK2-STAT3 pathway and the expression of ACSL4	Lipid peroxidation and ROS production	Song et al. (2022)
Pseudolaric acid B	*Pseudolarix amabilis* (J.Nelson) Rehder (Pinaceae)	Glioma	Improving intracellular iron by upregulation of transferrin receptor and then activating NOX4	Increasing the accumulation of H2O2 and lipid peroxides	Wang et al. (2018)
Brucine	*Strychnos nux-vomica* L. (Loganiaceae)	Glioma	Inducing ATF3 upregulation and translocation into nuclei via activation of ER stress	Promoting lipid peroxides and intracellular chelate iron enrichment	Lu et al. (2021)
Artesunate	Artemisinin; *Artemisia annua* L. (Asteraceae)	Head and neck cancer cells	NRF2-ARE pathway activation	Decreasing cellular GSH levels and increasing lipid ROS levels	Roh et al. (2017)
Dihydroartemisinin	Artemisinin; *Artemisia annua* L. (Asteraceae)	Head and neck cancer cells	Downregulating the expression of GPX4	Increasing ROS accumulation	Lin et al. (2016)
Piperlongumine	*Piper longum* L. (Piperaceae)	Head and neck cancer cells	Targeting the stress response to ROS, leading to the induction of death pathways involving JNK and PARP	Increasing ROS accumulation	Roh et al. (2014)
Tanshinone IIA	*Salvia miltiorrhiza Bunge* (Lamiaceae)	Head and neck cancer cells	Suppressing FTH1 expression	Dysregulation of iron homeostasis	Mao et al. (2022)
Cucurbitacin B	*Trichosanthes kirilowii* Maxim. (Cucurbitaceae)	Head and neck cancer cells	Downregulating the expression of GPX4	Lipid peroxidation	Huang et al. (2021)
Polyphyllin II	*Paris yunnanensis* Franch. (Melanthiaceae)	Hepatocellular carcinoma	Depending on NCOA4 and FTH1 expression	Ferritinophagy	Lin et al. (2020)
Solasonine	Solanum melongena L. (Solanaceae)	Hepatocellular carcinoma	Inhibiting GPX4 and GSS	Inducing ROS production	Jin et al. (2020)
6-Gingerol	*Zingiber officinale* Roscoe (Zingiberaceae)	Lung cancer	Decreasing the expression of USP14	Increasing ROS and iron concentration	Tsai et al. (2020)
Andrographolide	*Andrographis paniculata* (Burm.f.) Nees (Acanthaceae)	Lung cancer	Inhibitingthe expression of GPX4 and SLC7A11	Inducing ROS production	Li et al. (2023a)
Curcumenol	*Curcuma aromatica* Salisb. (Zingiberaceae)	Lung cancer	lncRNA H19/miR-19b-3p/FTH1 pathway	Inducing ROS generation, GSH depletion, lipid peroxidation, and iron accumulation	Zhang et al. (2022)
Dihydroartemisinin	Artemisinin; *Artemisia annua* L. (Asteraceae)	Lung cancer	downregulating Cystine/glutamate transporter and upregulating TFRC	Increasing ROS and iron concentration	Zhang et al. (2021b)
Epigallocatechin-3-Gallate	*Camellia sinensis* (L.) Kuntze (Theaceae)	Lung cancer	Targeting STAT1/SLC7A11 pathway	Inducing ROS production	Li et al. (2023b)
Erianin	*Dendrobium chrysotoxum* Lindl. (Orchidaceae)	Lung cancer	Downregulating GPX4 and SLC7A11; Ca2+/CaM signaling dependent manner	ROS accumulation, lipid peroxidation, and GSH depletion	Zhu et al., 2019
Ginkgetin	*Ginkgo biloba* L. (Ginkgoaceae)	Lung cancer	Activation of NRF2/HO-1	Increasing labile iron pool and lipid peroxidation	Lou et al. (2021)
β-elemene	*Curcuma longa* L. (Zingiberaceae)	Lung cancer	Upregulating lncRNA H19	Inducing ROS production	Xu et al. (2023a)
Brusatol	*Brucea javanica* (L.) Merr. (Simaroubaceae)	Lung cancer	NRF2-FOCAD-FAK signaling pathway with cysteine deprivation	Inducing ROS production	Liu et al. (2020a)
Hedyotis diffusa injection	*Scleromitrion diffusum* (Willd.) R.J.Wang (Rubiaceae)	Lung cancer	Bcl2 inhibition to promote Bax regulation of VDAC2/3	Lipid peroxidation and ROS production	Huang et al. (2022)
Red ginseng polysaccharide	*Panax ginseng* C. A. Meyer (Araliaceae)	Lung cancer	Suppressing the expression of GPX4	Lipid peroxidation and ROS production	Zhai et al. (2022)
Baicalein	*Scutellaria baicalensis* Georgi (Lamiaceae)	Myeloid leukemia	Inhibiting the protein expression of GPX4	Lipid peroxidation and ROS production	Probst et al. (2017)
Dihydroartemisinin	Artemisinin; *Artemisia annua* L. (Asteraceae)	Myeloid leukemia	Regulating the activity of AMPK/mTOR/p70S6k signaling pathway	Accelerating the degradation of ferritin, increased the labile iron pool, promoted the accumulation of cellular ROS	Du et al. (2019)
Honokiol	*Magnolia officinalis* Rehder and E.H.Wilson (Magnoliaceae)	Myeloid leukemia	Upregulating the level of intracellular lipid peroxide and HMOX1	Triggering a noncanonical ferroptosis pathway	Lai et al. (2022)
Curcumin	*Curcuma longa* L. (Zingiberaceae)	Ovarian cancer	Inhibiting the protein expression of GPX4	Lipid peroxidation and ROS production	Shi et al. (2023a)
Dihydroartemisinin	Artemisinin; *Artemisia annua* L. (Asteraceae)	Ovarian cancer	Decreasing GSH and GPX4 activity	ROS-dependent DNA damage and cell death	Greenshields et al. (2017)
Artesunate	Artemisinin; *Artemisia annua* L. (Asteraceae)	Pancreatic cancer	Decreasing GSH and GPX4 activity	Lipid peroxidation and ROS production	Eling et al. (2015)
Baicalein	*Scutellaria baicalensis* Georgi (Lamiaceae)	Pancreatic cancer	Degradation of glutathione peroxidase 4	Lipid peroxidation	Xie et al., 2016 Zhou et al. (2017)
Piperlongumine	*Piper longum* L. (Piperaceae)	Pancreatic cancer	GPX4 inactivation	ROS production	Yamaguchi et al. (2018)
Brusatol	*Brucea javanica* (L.) Merr. (Simaroubaceae)	Pancreatic cancer	Inhibiting the activation of NRF2 defense pathway under hyperoxidation and hyperthermia and causing GPX4 and FTH inactivation	Lipid peroxidation and ROS production	Tao et al. (2022)
Curcumol	*Curcuma longa* L. (Zingiberaceae)	Prostate cancer	miR-7/mTOR/SREBP1 pathway	ROS production	Xu et al. (2023b)
Cariin	*Epimedium sagittatum* (Siebold and Zucc.) Maxim. (Berberidaceae)	Prostate cancer			
Artesunate	Artemisinin; *Artemisia annua* L. (Asteraceae)	Prostate cancer	_	ROS production	Vakhrusheva et al. (2022)
Curcumin	*Curcuma longa* L. (Zingiberaceae)	Renal carcinoma	Increasing the expression of ADAMTS18	Inhibiting expression of NCOA4 and FTH1	Xu et al. (2021)
Curcumin	*Curcuma longa* L. (Zingiberaceae)	Thyroid Cancer	Upregulating HO-1 expression	Lipid peroxidation induced by GPX4 inhibition	Chen et al. (2023a)

## 6 Conclusion

In summary, the accumulation of lipid peroxide due to excessive ROS, exceeding the antioxidant enzyme clearance capacity, promotes ferroptosis, and iron is required for this process. The regulation of ferroptosis is complex and primarily involves pathways related to the metabolism of amino acids, iron, and lipids. Abundant metabolism-associated regulatory mechanisms make the targeting of ferroptosis a promising strategy for cancer treatment. Recently, TCMs have garnered considerable interest owing to their natural origins and limited side reactions. Additionally, plant metabolites and derivatives can regulate ferroptosis by targeting SLC7A11, GPX4, and ACSL4 and influence the immune status in the tumor microenvironment, providing new insights and directions for TCMs in cancer treatment.
